# Plasma peroxiredoxin changes and inflammatory cytokines support the involvement of neuro-inflammation and oxidative stress in Autism Spectrum Disorder

**DOI:** 10.1186/s12967-019-2076-z

**Published:** 2019-10-02

**Authors:** P. M. Abruzzo, A. Matté, A. Bolotta, E. Federti, A. Ghezzo, T. Guarnieri, M. Marini, A. Posar, A. Siciliano, L. De Franceschi, P. Visconti

**Affiliations:** 1 Department of Experimental, Diagnostic and Specialty Medicine, University of Bologna School of Medicine, Bologna, Italy; 2 IRCCS Fondazione Don Carlo Gnocchi, Via A. Capecelatro, 66, 20148 Milan, Italy; 3 Department of Medicine, University of Verona Medical School, Verona, Italy; 4 Department of Biological, Geological and Environmental Sciences, University of Bologna, Bologna, Italy; 5 Department of Biomedical and Neuromotor Sciences, University of Bologna, Via Ugo Foscolo 7, 40123 Bologna, Italy; 6 Child Neurology and Psychiatry Unit, IRCCS Istituto delle Scienze Neurologiche di Bologna, Via Altura, 3, 40139 Bologna, Italy

**Keywords:** Autism Spectrum Disorder, Peroxiredoxins 1, 2, 3 and 5, Red cells, Oxidative stress, (Neuro)inflammation, Cytokines, AHR signaling pathway

## Abstract

**Background:**

It has been established that children with Autism Spectrum Disorders (ASD) are affected by oxidative stress, the origin of which is still under investigation. In the present work, we evaluated inflammatory and pro-oxidant soluble signature in non-syndromic ASD and age-matched typically developing (TD) control children.

**Methods:**

We analyzed leukocyte gene expression of inflammatory cytokines and inflammation/oxidative-stress related molecules in 21 ASD and 20 TD children. Moreover, in another—comparable—group of non-syndromic ASD (N = 22) and TD (N = 21) children, we analyzed for the first time the protein expression of the four members of the antioxidant enzyme family of peroxiredoxins (Prx) in both erythrocyte membranes and in plasma.

**Results:**

The gene expression of IL6 and of HSP70i, a stress protein, was increased in ASD children. Moreover, gene expression of many inflammatory cytokines and inflammation/oxidative stress-related proteins correlated with clinical features, and appeared to be linked by a complex network of inter-correlations involving the Aryl Hydrocarbon Receptor signaling pathway. In addition, when the study of inter-correlations within the expression pattern of these molecules was extended to include the healthy subjects, the intrinsic physiological relationships of the inflammatory/oxidative stress network emerged. Plasma levels of Prx2 and Prx5 were remarkably increased in ASD compared to healthy controls, while no significant differences were found in red cell Prx levels.

**Conclusions:**

Previous findings reported elevated inflammatory cytokines in the plasma of ASD children, without clearly pointing to the presence of neuro-inflammation. On the other hand, the finding of microglia activation in autoptic specimens was clearly suggesting the presence of neuro-inflammation in ASD. Given the role of peroxiredoxins in the protection of brain cells against oxidative stress, the whole of our results, using peripheral data collected in living patients, support the involvement of neuro-inflammation in ASD, and generate a rational for neuro-inflammation as a possible therapeutic target and for plasma Prx5 as a novel indicator of ASD severity.

## Background

Autism Spectrum Disorder (ASD) is a heterogeneous group of pervasive neurodevelopmental disorders characterized by impairment in the areas of communication, social behavior, and restricted interests and activities [1–3].

ASD has a strong genetic basis [4, 5], with over 100 identified monogenic syndromes [6], a high number of susceptibility genes, of copy number variation (CNV) loci, and of rare genetic mutations/variants; however, genetics alone accounts to only 30–35% of ASD cases [7]. Indeed, despite the high concordance among homozygous twins, a “broader autism phenotype” can be recognizable, though in a milder form, in the majority of siblings and parents of ASD subjects [8], thus suggesting the presence of still unidentified molecular defects and the interplay with environmental factors. In fact, environmental factors, as diverse as exposure to toxicants, composition of the enteric microbiota, and immune dysregulation [9–11], have been suggested to play a role in the establishment of the autistic phenotype, thus accounting for the remaining 65–70% of ASD cases. By affecting epigenetic modifications altering gene expression levels [12], they are supposed to interact with a still ill-defined “susceptible” genome.

Thus, non-syndromic ASD appears as a complex genetic trait, resulting from the combination of multiple de novo mutations, CNV, rare genetic variants and epigenetic regulation, with possible additive effects. Such etiological complexity forms the basis of the heterogeneity of the disease and challenges the attempts of finding therapeutic solutions.

Since social communication, skills and range of interests and activities are the most affected areas in ASD subjects, researchers primarily focus on the identification of mutations impairing the correct neuronal functioning of central nervous system (CNS). As a matter of fact, a significant number of potential ASD candidate genes is involved in processes of cell-to-cell communication between neurons or between neural and microglial cells. However, being oxidative stress and inflammation present in ASD children, the possible contribution of neuroinflammation in the pathogenesis of ASD has recently gained growing attention [13]. This has been also invocated in a number of psychiatric disorders, such as major depressive disorder, bipolar disorder, and schizophrenia, even though the exact underlying mechanisms are still lacking [14]. The analysis of post-mortem brain from ASD subjects suggests that microglia activation or dysfunction may be itself caused by an increased inflammatory response and oxidation [15–17]. These findings, together with the recent evidence of an increase in soluble pro-inflammatory cytokines in blood and cerebrospinal fluid in ASD patients, support the involvement of neuroinflammation in the pathogenesis of ASD [16–20]. Indeed, children with ASD bear hallmarks of abnormalities in systemic redox balance [21–24] as well as of increase in soluble makers of inflammation [25, 26]. Using red cells as cell model to explore oxidative stress in ASD, we reported abnormal red cell membrane lipid composition, reflecting increased pro-oxidant environment in ASD subjects [23], and, more recently, the presence of advanced glycation end-products in plasma and urine of ASD children and the up-regulation of pro-inflammatory and anti-oxidant systems in isolated polymorphonuclear cells [27, 28].

Peroxiredoxins (Prxs) are ubiquitous anti-oxidants with a multifunction profile, acting as peroxidase scavengers but also as atypical molecular chaperones [29–35]. Prxs might be also released by different cell types in response to inflammatory stimuli or oxidation, contributing to the modulation of the cytokine storm [36, 37]. Neurons express Prx2 and 5, similarly to other cells such as erythrocytes, adipocytes or cardiomyocytes [30, 38–41]. In a mouse model of brain ischemia, Prx2 and/or Prx5 have been also detected outside brain cells, contributing to the modulation of microglia and to the local modulation of inflammatory response [34]. In addition, treatment with Anti-Prx5 antibody after the ischemic events beneficially affects the infarct size and the mouse neurologic outcomes [34]. These findings are in agreement with previous studies in both cell-based and animal-based systems, showing the importance of Prxs in neuronal homeostasis and in cell defense against oxidation [38, 42, 43]. Noteworthy, increased expression of Prx5 has been reported in psychiatric disorders such as schizophrenia or bipolar disorder [44, 45], whereas Prx2 has been found to be up-regulated in frontal cortex of subjects with neurodegenerative disorders including Down syndrome [46].

Here, we compared red cell membrane and plasma levels of Prx in ASD and typically developing (TD) children and show that plasma Prx2 and Prx5 correlate with ASD. Moreover, we analyzed the gene expression network of inflammatory cytokines and inflammation/oxidative stress-related proteins and its relation with clinical features, which led us to suggest the protective role played by plasma Prxs and the involvement of the Aryl Hydrocarbon Receptor signaling pathway in ASD children.

## Methods

### Ethics statement

The present study was conducted according to the guidelines laid down in the Declaration of Helsinki and all procedures were approved by Local Ethical Committee (Azienda USL Bologna, CE 10020-n.30, 06/04/2010 prot. 45424/10-03 and CE 13062, 23/12/2013; prot. N.1198/CE). Written consent was obtained from all parents and, whenever possible, also from children through pictures and simplified information.

### Subjects

Two highly comparable groups of children were studied. The first group was the source of leukocytes for RNA extraction and evaluation of the gene expression. It was composed of 21 children diagnosed with non-syndromic ASD children [17 males and 4 females, aged (mean ± SD) 6.8 years] and 20 TD children [14 males and 6 females, aged (mean ± SD) 7.6 years]. Demographic parameters and clinical features of this group have been published by Ghezzo et al. [23]. The second group was made up of 22 non-syndromic ASD children (17 males and 5 females), aged (mean ± SD) 7.84 ± 1.86 years and 21 TD children [14 males and 7 females, aged (mean ± SD) 9.47 ± 2 years]. Demographic parameters and clinical features of this group have been published by Bolotta et al. [28]. Blood samples from this group were the source of erythrocytes and plasma for the evaluation of Prx levels. Within each group, both the non-parametric comparison of the average age of ASD and TD and the comparison by gender (Chi-square test) were not significant, confirming the comparability between cases and controls. All children were recruited by the Child Neuropsychiatry Unit of the Bellaria Hospital (IRCCS, Bologna) within the local community. The patients underwent a clinical diagnostic assessment and a comprehensive neurological work up, including electroencephalography (recorded both awake and sleeping), cerebral magnetic resonance imaging, CGH Array and molecular assay for Fragile X and MECP2. Childhood Autism Rating Scale (CARS) total scores ranged from mild to severe autistic features; developmental scores varied from normal IQ to severe cognitive impairment. Control TD children were recruited in the same local community and were free of cognitive, learning and psychiatric problems. All subjects did not take any dietary supplement in the 4 months preceding the biochemical and clinical evaluations and were free of any inflammatory or infective problems.

### Blood samples and their handling

Blood samples were collected in Na_2_-EDTA vacutainers. Basal hematological parameters were examined by routine laboratory techniques. Plasma was obtained by centrifugation. Peripheral blood mononuclear cells (PBMC) were separated by a discontinuous Ficoll density gradient.

### RNA extraction, cDNA synthesis and RT-PCR analysis

PBMCs were lysed in 1 mL Trizol^®^ Reagent (Invitrogen, Italy) and RNA was extracted, quality controlled and reverse transcribed as previously described in detail [47]. Quantitative Real-Time PCR (qRT-PCR) was performed by a CFX96 real-time thermal cycler using the SsoAdvanced™ Universal SYBR^®^ Green Supermix (Biorad, USA). All primers used in this study (Table 1) were custom designed with the help of the Primer Blast, Primer3 and AMPLIFY free software; whenever possible, primers were designed so as to span an exon–exon junction. Primers were obtained from GENOSYS (Sigma, USA). For normalization purposes, the expression of the housekeeping genes Actin-beta and GAPDH was quantified. Data were analyzed by using the CFX Manager and the qBase software and expressed as means ± confidence interval.

**Table 1:** Primer sequences and amplicon length of the genes studied by qRT-PCR

Unigene accession no.	Gene ID	Gene	Left primer	Right primer	Amplicon length (bp)
Hs.171189	196	AHR	CTTCCAAGCGGCATAGAGAC	AGTTATCCTGGCCTCCGTTT	198
Hs.274402	3303	HSPA1A^a,b^	CCCTGATCAAGCGCAACT	CTCGTACACCTGGATCAGCA	98
Hs.654458	3569	IL6	CAATGAGGAGACTTGCCTGGT	AGCTGCGCAGAATGAGATGA	275
Hs.193717	3586	IL10	GGCGCTGTCATCGATTTCTTC	GCCACCCTGATGTCTCAGTT	181
Hs.126256	3553	IL1 beta	AGCCATGGCAGAAGTACCTG	CCTGGAAGGAGCACTTCATCT	116
Hs.673	3592	IL12A	GCTCCAGAAGGCCAGACAAA	GCCAGGCAACTCCCATTAGT	183
Hs.201978	5742	PTGS1^b^	AGCTCGTAGGAGAGAAGGAGAT	AGTGTGGCCGTCTTGACAAT	253
Hs.196384	5743	PTGS2^b^	CAAATTGCTGGCAGGGTTGC	AGGGCTTCAGCATAAAGCGT	139
Hs.432121	7001	PRDX2	ACGAGCATGGGGAAGTTTGT	GCCTTTCCTGGGTCAGCATA	201
Hs.502823	25824	PRDX5	TCTTTGGGAATCGACGTCTC	ATTGCAGAAATCTGGCCAAC	229
Hs.463059	6774	STAT3	CGGAGAAGCATCGTGAGTGA	CTCTTCCAGTCAGCCAGCTC	95
Hs.241570	7124	TNF-alpha	CCCCAGGGACCTCTCTCTAA	TCTCAGCTCCACGCCATT	172
Hs.520640	60	Actin-beta^c^	TGTGGCATCCACGAAACTAC	TGATCTTGATCTTCATTGTGCT	175
Hs.544577	2597	GAPDH^c^	GGCCTCCAAGGAGTAAGACC	CTGTGAGGAGGGGAGATTCA	130

^a^These primers recognize also HSPA1B, a minor isoform of HSPA1A

^b^HSPA1A, protein name HSP70, inducible form; PTGS1 and PTGS2, protein name COX1 and COX2 (CICLOOXYGENASE 1 AND 2)

^c^Actin-beta and GADPH were the housekeeping genes utilized in the gene expression study

### Red cell membrane ghost preparation

Blood was centrifuged at 3000*g* for 5 min at 4 °C to remove plasma, passed through cotton to remove white cells, and washed three times with choline wash solution (CWS: 175 mM choline, 1 mM MgCl_2_, 10 mM Tris-MOPS pH 7.4 at 4 °C, 320–340 mOsm) [48]. Packed red cells were lysed in phosphate lysis buffer (PLB: 5 mM Na_2_HPO_4_ pH 8.0, added of a protease inhibitor cocktail tablet, 3 mM benzamidine, 1 mM Na_3_VO_4_ final concentration) and washed in PLB 5 times to obtain almost white ghosts. Whenever Prx2 was evaluated in SDS-PAGE analysis, 100 mM of NEM was added to the PLB to avoid possible artifacts due to Prx2 oxidation after cell lysis [29–33, 49].

### Immunoblot analysis of red cell membrane and plasma

Mono-dimensional electrophoresis was carried out as previously described [50, 51]. Gels were transferred to nitrocellulose membranes for immuno-blot analysis with specific antibodies: anti-peroxiredoxin-1 (Prx1, polyclonal Ab, Abcam, UK), anti-peroxiredoxin-2 (Prx2, clone 1E8, Abcam, UK), anti-peroxiredoxin-3 (Prx3, polyclonal Ab, Abcam, UK) and anti-peroxiredoxin-5 (Prx5, clone 3F11, Abcam, UK); Actin (anti-actin; Sigma Aldrich, USA) and anti-IgG were used as loading controls. Secondary anti-rabbit IgG and anti-mouse IgG HRP conjugated were from GE Healthcare. Blots were developed using the chemiluminescence reagent Luminata HRP Chemiluminescence detection reagents. Densytometric analysis of band intensities was carried out using Quantity One analysis software (Bio-Rad, USA).

### Statistics

Normality tests were applied to all numeric variables, following which appropriate parametric tests (ANOVA, Student’s t for independent data) or the nonparametric equivalent (Wilcoxon-Mann–Whitney) were used to compare ASD and TD data. RT-PCR data are expressed as means ± confidence interval, where a significance level of 0.05 corresponds to the 95% confidence level. Non-parametric correlation (Spearman’s rho) was used to correlate clinical features and biochemical data in the ASD group (non-parametric ANOVA for cognitive/developmental level). Differences were considered significant at p < 0.05. To account for multiple testing we used the Benjamini and Hochberg false discovery rate (FDR). FDR corrected p-values (pFDR) were evaluated separately for (a) correlations between gene expression of cytokines and inflammation/oxidation-related molecules and clinical features; (b) inter-correlations between gene expression of cytokines and inflammation/oxidation-related genes; (c) correlations of peroxiredoxins (proteins) with clinical features and (d) inter-correlations of peroxiredoxins (proteins).

## Results

### Pro-inflammatory signature characterizes peripheral blood mononuclear cells from ASD patients

Gene expression of cytokines and inflammation/oxidation-related molecules was examined in PBMC from the first group of ASD and TD children. For each gene studied, Table 2 reports ASD/TD ratio (± confidence interval), as well as the result of correlation tests with clinical features.

**Table 2:** Cytokines and inflammation/oxidation-related genes in leukocytes of ASD and TD children

Inflammation/oxidation-related genes	Fold change (ratio ASD/TD)	95% confidence interval (CI)	Two-sided *p* value for ASD vs TD difference	Correlation with total CARS score	Correlation with hyperactivity	Correlation with stereotypies	Correlation with cognitive level
AHR	0.77	0.471–1.281	NS	p = 0.0025 r = 0.70 pFDR = 0.12	NS	NS	NS
HSP70i	1.787	1.227–2.602	p < 0.01	NS	NS	NS	NS
IL6	2.204	1.246–3.898	p < 0.01	NS	NS	NS	NS
IL10	1.187	0.690–2.043	NS	p = 0.0246 r = 0.55 pFDR = 0.17	p = 0.0128 r = 0.6 pFDR =0.12	NS	p = 0.0377 r = 0.49 pFDR = 0.23
IL12A	1.537	0.893–2.646	NS	p = 0.0043 r = 0.67 pFDR = 0.13	NS	p = 0.0197 r = 0.58 pFDR = 0.16	NS
IL1 beta	1.339	0.779–2.303	NS	p = 0.007 r = 0.64 pFDR = 0.11	NS	NS	NS
COX1	1.025	0.785–1.337	NS	NS	NS	NS	NS
COX2	1.312	0.788–2.184	NS	p = 0.0435 r = 0.51 pFDR = 0.21	NS	NS	NS
PRX2	1.038	0.867–1.243	NS	NS	NS	p = 0.0111 r = 0.62 pFDR = 0.13	NS
PRX5	0.983	0.884–1.092	NS	NS	NS	NS	NS
STAT3	0.952	0.382–2.376	NS	NS	NS	NS	NS
TNF-alpha	0.999	0.567–1.759	NS	NS	NS	NS	NS

Statistically significant correlations are underlined

*ASD* Autism Spectrum Disorders, *TD* typically developing, *CARS* Childhood Autism Rating Scale, *AHR* Aryl Hydrocarbon Receptor, *HSP70i* Heat Shock Protein 70, inducible form, *IL* interleukin, *COX* cyclooxygenase, *PRX* peroxiredoxin, *STAT3* Signal Transducer and Activator of Transcription 3, *TNF-alpha* Tumor Necrosis Factor-alpha, *NS* not significant, *r* correlation coefficient

Comparisons between the ASD and the TD group of children revealed significant differences in the leukocyte expression of IL6, a major inflammatory cytokine, and of HSP70i, perhaps the most important chaperon involved in cellular defense mechanisms. Moreover, the gene expression of some inflammation/oxidation related genes (namely AHR, IL10, IL12A, IL1 beta, COX2 and PRX2) positively correlated with some ASD clinical features, although, after applying the False Discovery Rate test, correlations lost statistical significance. Remarkably, the gene expression of some cytokines and inflammation-related proteins was inter-correlated, thus suggesting a possible functional crosstalk within this pro-inflammatory network (Table 3). Again, the application of the False Discovery Rate test caused some of the inter-correlations to lose statistical significance. Of note, PRX5 expression inversely correlated with that of AHR, of COX2 and STAT3. These findings support a pro-inflammatory environment characterizing ASD patients and requiring efficient cytoprotective systems such as peroxiredoxins. The existence of inter-correlations within the gene expression of cytokines and inflammation-related products was put into test in the whole set of studied subjects belonging to the first group, i.e. both ASD and TD children. Half the inter-correlations within the gene expression of some cytokines and inflammation-related products were a general feature, not restricted to ASD patients. PRX5 expression inversely correlated with that of COX2 and STAT3 also in the general population, thus enforcing the concept of its role in inflammatory contexts.

**Table 3:** Correlations between gene expression of cytokines and inflammation/oxidation-related molecules

Cytokine or inflammation-related molecules	AHR	HSP70i	IL6	IL10	IL12A	IL1 beta	COX1	COX2	PRX2	PRX5	STAT3	TNF-alpha
AHR				p = 0.0014 r = 0.75 pFDR = 0.031		p = 0.0265 r = 0.56 pFDR = 0.159		p = 0.0458 r = 0.51 pFDR = 0.178		p = 0.0281 r = − 0.55 pFDR = 0.154	p = 0.0027 r = 0.70 pFDR = 0.036	p = 0.0417 r = 0.51 pFDR = 0.183
HSP70i			p = 0.0176 r = − 0.58 pFDR = 0.129		p = 0.0161 r = − 0.59 pFDR = 0.132							
IL6					p = 0.0079 r = 0.64 pFDR = 0.075							
IL10	*p* = *0.0112* *r* = *0.44* *pFDR* = *0.082*		*p* = *0.0484* *r* = *0.35* *pFDR* = *0.213*			p = 0.0012 r = 0.73 pFDR = 0.040						p = 0.0010 r = 0.74 pFDR = 0.066
IL12A		*p* = *0.0346* *r* = − *0,37* *pFDR* = *0.19*	*p* < *0.0001* *r* = *0.63* *pFDR* = *0.003*	*p* = *0.0049* *r* = *0.48* *pFDR* = *0.064*		p = 0.0059 r = 0.65 pFDR = 0.065						
IL1 beta		*p* = *0.006* *r* = − *0.47* *pFDR* = *0.057*	*p* = *0.0053* *r* = *0.47* *pFDR* = *0.058*									p = 0.0015 r = 0.73 pFDR = 0.025
COX1											p = 0.0420 r = 0.65 pFDR = 0.173	
COX2										p = 0.0320 r = − 0.69 pFDR = 0.162		
PRX2												
PRX5		*p* = *0.0368* *r* = − *0.36* *pFDR* = *0.187*						*p* = *0.0014* *r* = − *0.53* *pFDR* = *0.023*			p = 0.0386 r = − 0.52 pFDR = 0.182	
STAT3	*p* < *0.0001* *r* = *0.69* *pFDR* = *0.007*			*p* = *0.0176* *r* = *0.41* *pFDR* = *0.116*				*p* = *0.0484* *r* = *0.35* *pFDR* = *0.213*		*p* = *0.0221* *r* = − *0.40* *pFDR* = *0.133*		p = 0.0196 r = 0.58 pFDR = 0.129
TNF-alpha	*p* = *0.0194* *r* = *0.40* *pFDR* = *0.128*	*p* = *0.0435* *r* = − *0.35* *pFDR* = *0.205*		*p* = *0.0002* *r* = *0.60* *pFDR* = *0.004*							*p* = *0.0092* *r* = *0.45* *pFDR* = *0.076*	

Significant correlations (p ≤ 0.05) are shown. Correlations found in ASD children alone are shown in underline; correlations found in the whole population (ASD and TD children) are shown in italic

#### Prx2 and Prx5 significantly increase in plasma from ASD patients

**Fig. 1:**
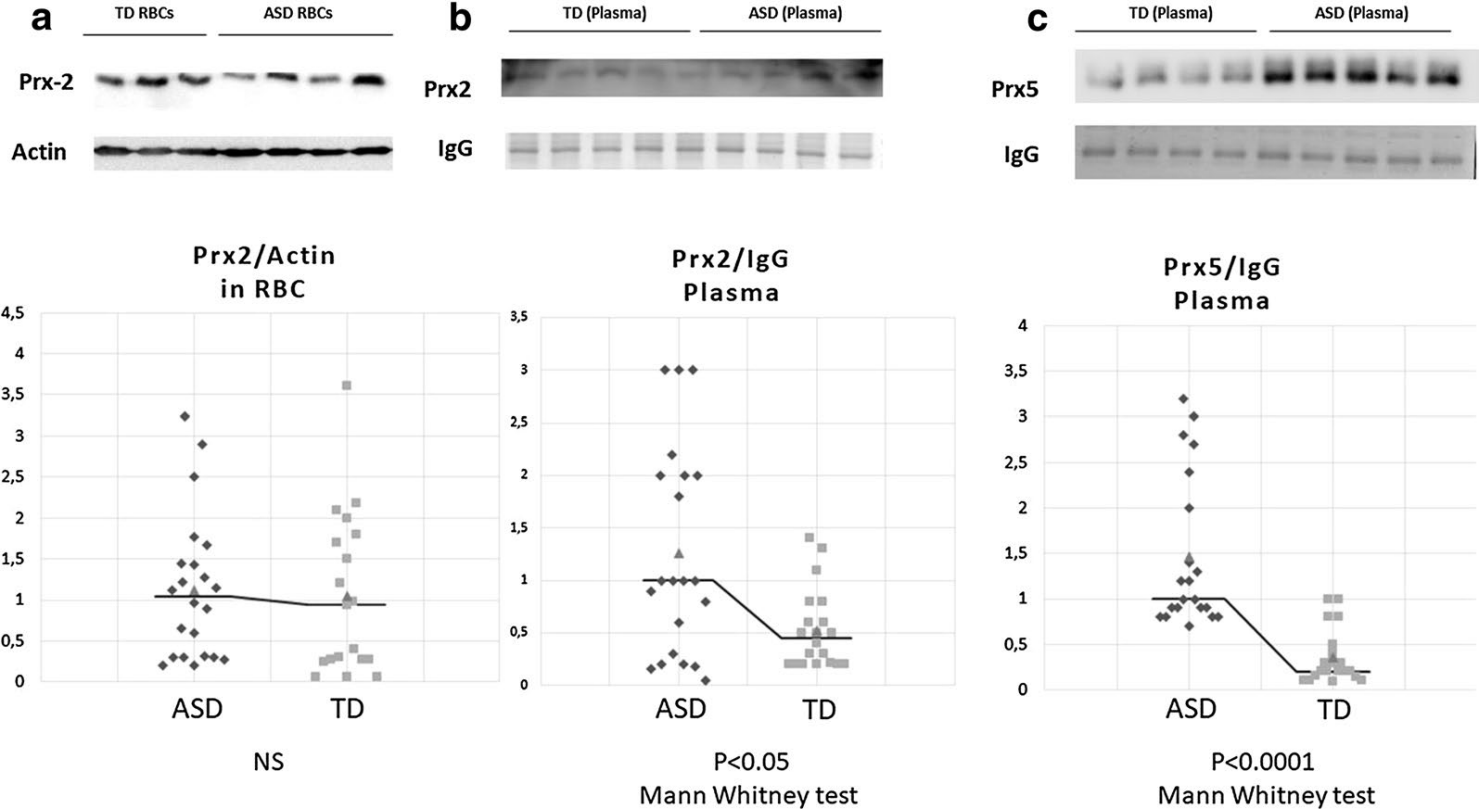
Upper panels. Immunoblot analysis with specific antibodies against **a** peroxiredoxin-2 (Prx2) in red cell membrane; **b** peroxiredoxin-2 (Prx2) in plasma; **c** peroxiredoxin-5 (Prx5) in plasma. Healthy controls (TD) and subjects with Autism Spectrum Disorder (ASD) were compared. Actin was used as loading control protein. One representative gel from three sets of separate experiments with similar results is presented. Lower panels. Densitometric analysis of immunoblots as in upper panels are shown as dot-plots

In the present study, we used red cells to evaluate Prx2 and Prx5 membrane association as a marker of membrane oxidation in ASD patients [29–33]. Prx2 and Prx5 are differently expressed in red cells, the former being more abundant than the latter [38, 52, 53]. As shown in Fig. 1a, no significant differences in Prx2 red cell membrane association in ASD patients was found in comparison with normal controls. Similar results were also obtained when Prx5 membrane translocation was studied in red cells from both groups of subjects (data not shown).

Since Prxs are released by different cell types in response to inflammation and might contribute to the release of pro-inflammatory cytokines [54], we evaluated Prx2 and Prx5 plasma levels in ASD and healthy controls. As shown in Fig. 1b, plasma Prx2 was significantly higher in ASD patients with respect to control subjects. In addition, plasma Prx5 was markedly increased in ASD patients when compared to healthy subjects. No major change in plasma Prx1 and Prx3 levels were observed in ASD patients compared to healthy controls (data not shown).

We then evaluated the relation between Prx2 red cell membrane translocation or Prx5 plasma levels and ASD clinical presentation. Table 4 shows that the amount of Prx2 associated to the red cell membrane, though not significantly increased in ASD compared to TD subjects, was nevertheless negatively correlated with some ASD clinical features; the same was true for the amount of Prx5 in the plasma. This underscores the protective role of Prxs, since it suggests that the ability of transferring Prx2 to the erythrocyte cell membrane and of releasing Prx5 in the plasma goes in hand with a milder ASD phenotype.

**Table 4:** Correlations of peroxiredoxins (protein amount) and clinical features

	Age (months)	Onset pattern: 1 (early); 2 (regressive); 3 (mixed)	Brief non verbal IQ*	ADOS score**	CARS total score	CARS Activity Level item score	CARS body use (sterotypes) item score	CARS verbal communication item score	Non verbal communication Item score	CARS total number of items whose score was ≥ 3	Prx2/IgG in plasma	Prx5/IgG in plasma
Prx2/actin in RBC	NS	NS	NS	NS	p = 0.027 r = − 0.47 pFDR = 0.13	p = 0.0043 r = − 0.58 pFDR = 0.13	NS	NS	p = 0.020 r = − 0.49 pFDR = 0.12	p = 0.01 r = − 0.5 pFDR = 0.15	NS	p = 0.0048 r = 0.59 pFDR = 0.01
Prx2/IgG in plasma	NS	NS	NS	NS	NS	NS	NS	NS	NS	NS		NS
Prx5/IgG in plasma	NS	NS	NS	p = 0.021 r = − 0.5 pFDR = 0.16	NS	NS	NS	NS	NS	p = 0.022 r = − 0.5 pFDR = 0.13		

*RBC* red blood cell, *Prx* peroxiredoxin, *IgG* immunoglobulin G, *ADOS* Autism Diagnostic Observation Schedule, *CARS* Childhood Autism Rating Scale

*Cognitive level: ≥ 70, normal; 50-69, mild cognitive impairment; 35-49, moderate cognitive impairment; < 35, severe cognitive impairment [55]

**ADOS modules 1 or 2 (Total score Autism cut off = 12)

#### Plasma Prx5 levels are suggestive of ASD

**Fig. 2:**
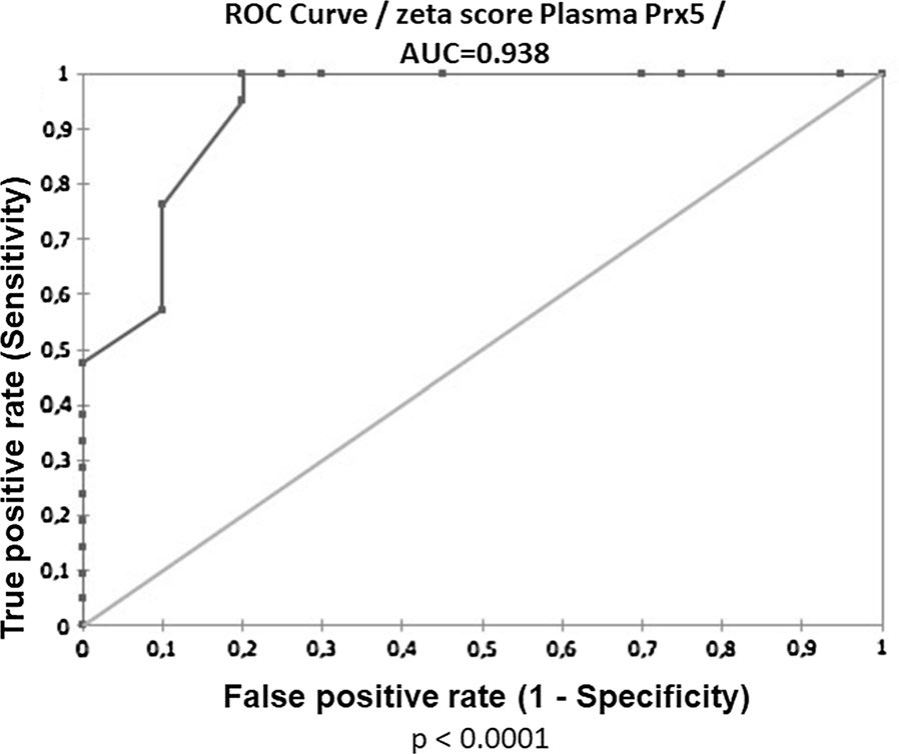
Receiver Operating Characteristic (ROC) curve showing that plasma Prx5 levels are able to identify ASD from TD subjects with high sensitivity and high specificity. The closer Area Under the Curve (AUC) is to 1.0, the higher are the sensitivity and the specificity of the comparison

Since the difference in Prx5 plasma levels between ASD and TD subjects was highly significant (p < 0.0001 by Mann–Whitney test), we calculated the Receiver Operating Characteristic (ROC) curve. The Area Under the Curve (AUC) value for plasma Prx5 resulted 0.938 (Fig. 2), thus suggesting that plasma Prx5 levels could be used to support ASD diagnosis with satisfactory indices of specificity and sensitivity.

## Discussion

Here, we show a pro-inflammatory signature in PBMCs from ASD patients and report for the first time the increase in plasma Prx5 levels. Although the source of Prx5 in the peripheral circulation is largely unknown, by reporting that Prx5 is involved not only in the local modulation of the inflammatory response but specifically in neuroprotection mechanisms [38, 45, 56–59], literature data are suggestive of the involvement of neuroinflammatory processes in ASD patients.

The main evidence of the involvement of CNS inflammation in ASD comes from autoptic studies carried out on ASD subjects, reporting microglial and astrocytic activation, in different brain areas [13, 15–20, 60, 61]. Remarkably, CNS inflammation might also account for the GABAergic/glutamatergic imbalance and the consequential glutamate excitotoxicity described in a group of ASD children [62]. Moreover, the fact that seizures associate with high frequency to autism made Theoharides and Zhang [63] suggest the association of ASD with neuroinflammation.

The second line of evidence is based on increase in plasma level cytokines, chemokines and prostaglandins in ASD children when compared with age-matched TD children [64–67]. In a subgroup of ASD children, independently of the gravity of clinical symptoms, we found increased expression of IL6. Noteworthy, IL6 is viewed to play a crucial role in the development and plasticity of CNS [68] and it has been described to be involved in maternal immune activation (MIA), which may contribute to the development of autism, again through the establishment of prenatal pro-inflammatory pathways (reviewed in [69]). IL6 has been linked to the pathogenesis of autism since it is involved in the homeostasis between neuro- and gliogenesis [70, 71] and is synergistically induced by xenobiotics [72], the kynurenine pathway [73, 74] and by the Aryl Hydrocarbon Receptor (AHR) [75].

In our study, we found that the gene expression of a number of cytokines and inflammation/oxidation-related molecules, though not significantly increased in ASD, was inter-correlated and correlated also with ASD severity. This observation led us to postulate the involvement of the AHR signaling pathway in ASD. By interacting with the xenobiotic response element (XRE), AHR is one of the major regulators of phase I target genes. AHR gene expression directly correlated with IL10, IL1 beta, COX2, STAT3 and TNF-alpha expression in the ASD leukocyte samples. Worth noting, the above-mentioned IL6 inducers may directly stimulate AHR [76, 77]; in turn, AHR signaling may also mediate anti-inflammatory responses through IL10 induction [78]. Table 3 also shows that some correlations between cytokine gene expression were independent from the subject’s disease status, thus underscoring the physiological inter-relationships of the inflammation-related pathways. For instance, AHR and STAT3 expression displayed a strong inter-correlation (p < 0.0001, pFDR 0.007) when all subjects (both TD and ASD children) were considered; this is in agreement with what reported by Stobbe-Maicherski et al. [79], who demonstrated that STAT3 binds to a STAT motif in AHR promoter, thus modulating AHR expression. Of note, AHR is a cellular biosensor, which is activated by a great variety of environmental molecules. As its activation can evoke an inflammatory outcome, its involvement in ASD could suggest the hypothesis of an environmental contribution in the pathogenesis of ASD. The intermediate products of phase I enzymes are postulated to serve as potential inducers of NRF2 pathway/phase II enzymes, which provide protection against oxidative stress. Notably, NRF2 gene expression was found to be increased in PBMC from ASD subjects [28]. This is of interest for ASD since the NRF2 pathway plays an important role in neuroprotection mechanisms [80].

An additional layer of complexity comes from the recent understanding of the role of AHR in the regulation of intestinal immunity [81]. The central and enteric nervous systems are integrated in what is dubbed the “brain-gut axis”, a bidirectional communication system which makes use of the neural, endocrine, immune, and metabolic pathways, and includes the role of the gut flora as a source of a wide range of neuroactive molecules, regulating a host of CNS activities, affecting health, well-being, behavior, immunity. The concept that altered communication between the gut microbiome and the brain may play an important role in human brain disorders has recently received considerable attention [82]; in particular it has been suggested to be a possible causative mechanism contributing to ASD pathogenesis [83, 84]. Notably, the increase in ASD children of inflammation markers, which may result by the additive effects of central brain neuroinflammation and specific alterations of the intestinal mucosa, has been recently described [26, 27, 84, 85]. Of note, the expression of the cytoprotective and anti-oxidant molecules HSP70i and PRX5 was *negatively* correlated with that of inflammatory cytokines such as IL6, IL12A, IL1 beta and COX2.

The novel observation reporting the increase in plasma Prx5 as characterizing signature in ASD patients might well be the third evidence of the occurrence of neuroinflammation in ASD, given the Prx involvement in neuronal homeostatic responses [38, 42, 43]. Prx5 might act as anti-oxidant, atypical molecular chaperone inside the cells and as modulator of local inflammatory response outside the cells [31, 36, 38, 42, 43, 86, 87]. Over-expression of Prxs has been reported in subjects with Down syndrome and in patients with neurodegenerative disorders such as Alzheimer or Parkinson disease, suggesting a neuroprotective role of Prxs in these disorders [44]. Noteworthy, increased plasma levels of Prx1 and 3 have been described in 20 patients with autism [88]. In our cohort, we did not find significant differences in plasma level for Prx1 and Prx3. This might be related either to the number of patients studied or to the methodologic approach. Rather, we report here a significant increase in plasma Prx2 and Prx5 levels in ASD children. Among Prxs, Prx5 has been shown to be specifically increased in brain from Alzheimer’s disease patients and to be released outside brain cells in response to local neuroinflammation during post-ischemic events [38]. The importance of Prx5 against neurotoxic damage is also supported by evidence of reduced severity of stroke-related neurologic damage in Prx5 treated mice [38]. Worth noting, according to our study, the area under the ROC curve indicates that Prx5 may be used as a biomarker for ASD. However, the comparison was made here with TD children, whereas bona-fide biomarkers should be able to aid in differential diagnosis with other neurodevelopmental disorders: to this purpose, further studies should be performed.

## Conclusions

Although the present study has some limitation such as its explorative profile and the small number of ASD patients studied, the plasma level of Prx5 emerges as a new and direct suggestion—obtained in living patients—of the involvement of neuroinflammation in the pathogenesis of ASD, which adds to evidences coming from autoptic data and from the evaluation of inflammation markers. It may be interesting to note that peripheral markers may, in this context, be used to track central nervous system abnormalities. Finally, these findings lend to the view that neuro-inflammation may become a possible therapeutic target in ASD children. Future studies should be designed to evaluate the clinical relevance of plasma Prx5 in a larger cohort of ASD subjects.

## Data Availability

The Authors declare that all original data are available for inspection and evaluation.

## Abbreviations

ADOS: Autism Diagnostic Observation Schedule
AHR: Aryl Hydrocarbon Receptor
ASD: Autism Spectrum Disorder
CARS: Childhood Autism Rating Scale
CNS: central nervous system
COX: cyclooxygenase
HS70i: Heat Shock Protein 70, inducible form
IgG: immunoglobulin G
IL: interleukine
PBMC: peripheral blood mononuclear cells
MIA: maternal immune activation
PRX: peroxiredoxin
RBC: red blood cells
STAT: Signal Transducers and Activators of Transcription (i.e. transcription factors)
TD: typically developing
TNF: tumor necrosis factor
